# Diabète insipide néphrogénique induit par le lithium au cours d’une intoxication aiguë: à propos d’un cas

**DOI:** 10.11604/pamj.2023.45.77.32130

**Published:** 2023-06-07

**Authors:** Sara Echater, Mohammed Hasnaoui, Evelyne Lechner

**Affiliations:** 1 Etablissement Public de Santé Ville Evrard, Neuilly sur Marne, France,; 2 Service de Psychiatrie, CHU Mohammed VI, Faculté de Médecine et de la Pharmacie, Université Mohammed I, Oujda, Maroc

**Keywords:** Diabète insipide néphrogénique, intoxication aiguë, lithium, insuffisance rénale aiguë, cas clinique, Nephrogenic diabetes insipidus, acute toxicity, lithium, acute renal failure, case report

## Abstract

En cas de déshydratation, le lithium peut entraîner une intoxication aiguë, ce tableau se manifeste principalement par des troubles neurologiques pouvant aller jusqu´au coma, des troubles digestifs, des troubles hydroélectrolytiques, des troubles cardiovasculaires. Un diabète insipide néphrogénique peut également survenir. Nous rapportons le cas d'une patiente suivie pour trouble bipolaire depuis 20 ans et traitée par lithium depuis 14 ans, qui a présenté un diabète insipide induit par le lithium lors d'une intoxication aiguë. Notre objectif est de souligner l´importance et la nécessiter d´une surveillance stricte de la lithémie surtout dans des situations favorisant la déshydratation.

## Abstract (en)

In case of dehydration, lithium can cause acute intoxication. This picture is mainly manifested by neurological disorders that can go as far as coma, digestive disorders, hydroelectrolytic disorders, and cardiovascular disorders. We report the case of a patient followed for bipolar disorder for 20 years and treated with lithium for 14 years and who presented an acute lithium intoxication resulting from a diabetes insipidus. Our objective is to underline the importance of good hydration and strict monitoring of lithium levels especially in situations favouring dehydration, notably the polyuria of diabetes insipidus.

## Introduction

Le lithium constitue un traitement de référence dans le trouble bipolaire [1]. Le diabète insipide néphrogénique (DIN) est une complication qui n´est pas exceptionnelle, il survient lors de l´exposition chronique aux lithium chez 5 à 20% des cas [2], cependant, des rares cas sont rapportés au cours d´intoxication aiguë au lithium, alors qu´il s´agit d´un facteur aggravant [3]. Nous en rapportons une observation.

## Patient et observation

**Information de la patiente:** patiente âgée de 53 ans, suivie pour trouble bipolaire depuis 20 ans et traitée depuis 14 ans par lithium (800 mg forme à libération prolongée) avec le valproate de sodium 500 mg, ayant comme antécédent une hypothyroïdie sous levothyroxine 25 μg et dystonie tardive post neuroleptique traitée par neurostimulation profonde mise en place il y a 16 ans, elle ne consommait pas des substances psychoactives. Hospitalisée en service de psychiatrie pour une rechute dépressive de sa maladie bipolaire.

**Résultats cliniques:** l´examen à l´admission trouve une patiente bien orientée dans le temps et l´espace et durant son hospitalisation, elle a présenté une parotidite, en rétrospective, il s´agissait d´un facteur de déshydratation, car la douleur et les signes d´inflammation empêchaient la patiente de boire de l´eau.

**Démarche diagnostique:** à l´admission le bilan rénal et l'ionogramme sanguin étaient normaux et la lithiémie était à 0,98 mmol/l (N= 0,8 à 1,2 pour la forme LP).

Quelques jours après, la patiente est devenue confuse, désorientée dans le temps et l´espace, l´examen retrouve une température à 37°C, tension artérielle à 90/60 mmHg, un pouls à 122 bat/min, GCS à 11/15, une rigidité musculaire avec majoration de ses tremblements, une sècheresse buccale, et un pli cutané. Devant ce tableau la patiente a été transférée aux urgences organiques et son bilan biologique initial a montré lithémie à 2.1 mmol/l, une natrémie à 146 mmol/l (N=135-145 mmol/l), kaliémie à 3.1 mmol/l (N=3.5-4.5 mmol/l), calcémie à 2.6 mmol/l (N=2.2 -2.6 mmol/l), urée à 6,68 mmol/l, créatinémie à 195 micromol/l, DFG à 28.30 ml/min/1.72 m^2^ (insuffisance rénale sévère).

**Intervention thérapeutique:** le lithium est arrêté, la patiente a bénéficié d´une une perfusion intraveineuse isotonique avec supplémentation potassique.

**Suivi et résultats:** après 48H son état neurologique s´est aggravé, elle est devenue très agitée nécessitant la mise en place d´une contention, avec une natrémie qui s´est augmentée à 175 mmol/l, une polyurie hypotonique est apparue avec une diurèse à 4l/j, une natriurèse à 14 mmol/24h (N=100 -300 mmol/24h), kaliurèse à 35 mmol/24h (50 et 100 mmol/l), osmolarité urinaire basse à 104 mOsmol/kg. Le test à la desmopressine était négatif. Nous avons diagnostiqué un diabète insipide néphrogénique induit par le lithium. Un traitement par l´amiloride 10-20 mg/j a été prescrit avec surveillance rapprochée de la lithémie, du ionogramme sanguin, de la fonction rénale et du volume urinaire, au bout d´une semaine la diurèse s´est normalisée avec correction des troubles hydro-électrolytiques et la lithémie s´est abaissée à 0,76 mmol/l. Après normalisation de la fonction rénale et l´ionogramme, on a repris le valproate de sodium seul.

**Perspective du patient:** le lithium a stabilisé mon trouble bipolaire pendant longtemps, mais ma dernière expérience était terrible heureusement que la prise en charge et les tests de laboratoire ont été rapides. On m'a bien expliqué mon état et j´ai compris que je dois changer le traitement par un autre thymorégulateur.

**Consentement du patient:** la patiente a donné son consentement et certains détails ont été supprimés de la description de ce cas pour en garantir l'anonymat.

## Discussion

Diabète insipide néphrogénique (DIN) est un effet secondaire fréquent qui survient au cours du traitement chronique par le lithium du fait de l´atteinte tubulaire, mais il est très rarement décrit au cours des intoxications aiguës [2]. Le lithium entraîne une résistance des cellules de tube collecteur à l´hormone antidiurétique (ADH) par inhibition de la protéine effectrice des récepteurs de l´ADH (aquaporine 2 et 3). Cette perturbation est responsable d´une incapacité du rein à concentrer les urines ce qui est à l´origine d´une polyurie qui peut dépasser 3L/j, le DIN est sans conséquence clinique, mais peut à travers la polyurie entraîner une déshydratation, qui est un facteur de diminution d´élimination de lithium et donc son augmentation sérique entrainant une intoxication aiguë [1].

Le tableau clinique de toxicité au lithium est très variable et la sévérité du tableau n´est pas nécessairement reliée à la lithémie, la symptomatologie clinique se caractérise principalement par des signes neurologiques: confusion, agitation motrice, myoclonies, hypertonie musculaire, hyper-réflexie ostéo-tendineuse, des manifestations extrapyramidale (rigidité, tremblements), changements du tracé de l´électroencéphalogramme (ECG), des convulsions voire le coma qui sont des signes de gravité et peuvent survenir et entraîner la mort.

Il s´y associe des troubles gastro-intestinaux type vomissements et diarrhées responsables d´une déshydratation globale et une insuffisance rénale aiguë. Le syndrome polyuro-polydipsique sévère est peu rapportée en cas d´intoxication aiguë au lithium [4], le plus souvent il s´agit d´un facteur déclenchant la toxicité en raison de la déshydratation qui entraîne une insuffisance rénale aiguë et l´incapacité d´éliminer le lithium, ceci a été rapporté dernièrement par Kobylianskii *et al*. [5], ainsi, l´existence d´une polyurie sévère constitue également un facteur aggravant qu´il faut prendre en compte en raison de la déshydratation et l´hypernatrémie qu´elle entraîne [3]. D’après nos connaissances et jusqu´à maintenant des rares cas ont été rapportés dans la littérature. Dans la série de Schmitt *et al*. quatre patients ont développé un véritable diabète insipide (diurèse supérieure à 3 L/24h). Tran-van *et al*. et Abodo *et al*. ont également rapporté deux cas d´un DIN survenu au cours d´une intoxication aiguë au lithium [2,3].

## Conclusion

Le DIN lié au lithium reste rare au cours des intoxications aiguës. Nous insistons sur l´importance d´une bonne hydratation et sur la nécessité d´une surveillance de la lithiémie surtout dans les circonstances favorisant une déshydratation.
